# Spatially resolved transcriptomics: advances and applications

**DOI:** 10.1097/BS9.0000000000000141

**Published:** 2022-11-04

**Authors:** Honglin Duan, Tao Cheng, Hui Cheng

**Affiliations:** a State Key Laboratory of Experimental Hematology, Haihe Laboratory of Cell Ecosystem, National Clinical Research Center for Blood Diseases, Institute of Hematology and Blood Diseases Hospital, Chinese Academy of Medical Sciences and Peking Union Medical College, Tianjin, China; b Center for Stem Cell Medicine, Chinese Academy of Medical Sciences, Tianjin, China; c Department of Stem Cell & Regenerative Medicine, Peking Union Medical College, Tianjin, China

**Keywords:** In situ hybridization, In situ sequencing, Spatially resolved multi-omics, Spatially resolved transcriptomics

## Abstract

Spatial transcriptomics, which is capable of both measuring all gene activity in a tissue sample and mapping where this activity occurs, is vastly improving our understanding of biological processes and disease. The field has expanded rapidly in recent years, and the development of several new technologies has resulted in spatially resolved transcriptomics (SRT) becoming highly multiplexed, high-resolution, and high-throughput. Here, we summarize and compare the major methods of SRT, including imaging-based methods, sequencing-based methods, and in situ sequencing methods. We also highlight some typical applications of SRT in neuroscience, cancer biology, developmental biology, and hematology. Finally, we discuss future possibilities for improving spatially resolved transcriptomic methods and the expected applications of such methods, especially in the adult bone marrow, anticipating that new developments will unlock the full potential of spatially resolved multi-omics in both biological research and the clinic.

## 1. INTRODUCTION

Omics technologies, which provide a comprehensive, global assessment of a set of molecules,^1^ offer a novel approach for the study of biological activity in life-sciences research. In recent years, the omics field has advanced considerably due to developments in the high-throughput analysis of biological molecules. Transcriptomic technologies in particular have provided rich insights into cellular characteristics, and measure the complete set of RNA transcripts produced by the genome under specific circumstances. Indeed, characterizing the transcriptome of individual cells is fundamental to improve our understanding of complex biological systems. Over the last decade, the rapid development of single-cell sequencing technology has greatly accelerated biomedical research, allowing scientists to overcome the major challenge of heterogeneity within biological samples and leading to the publication of a series of single-cell transcriptome atlases.^2–4^

However, most single-cell RNA sequencing (scRNA-seq) approaches involve isolating cells from their original position, meaning that spatial information is lost during transcriptome profiling. This omission makes it impossible to examine how a given cellular state interacts with neighboring cells or the surrounding extracellular matrix.^2,5,6^ To make up for this deficit, a new technology known as spatially resolved transcriptomics (SRT) aims to determine gene expression profiles while preserving information about the spatial context of the tissue. This technique is of great significance, since identifying the specific spatial location and organization of different cell types in multicellular tissues or organs is the foundation for research into cellular biological functions. Transcriptomics that is accompanied by spatial information will undoubtedly enable us to better understand the organization of cells and tissues, and how this organization influences biological function.^7^ The application of SRT has also opened up new areas of spatially resolved omics, including genomic, transcriptomic, proteomic and potentially other omic data with retained positional information. The ability of SRT to change the way we understand complex tissues earned it the title of *Method of the Year 2020* in Nature Methods.^8^ In this review, we will first summarize and compare the major methods of SRT (Table 1), highlight some current applications of SRT, and finally discuss our perspectives on the potential future applications of this method.

**Table 1:** Major SRT methods.

Major methods of spatially resolved transcriptomics				
Category	Method	Principle	Strengths	Limitations
smFISH	smFISH	In situ hybridization via complementary base pairing	High sensitivity Subcellular resolution Quantitative Without amplification bias	Low throughput
Multiplexed FISH	SRM	Optical SRM and combinatorial labeling	Improved throughput of smFISH	Low throughput
	ExFISH	Physical magnification	High-resolution imaging of RNA structure and location	Specific materials needed
	seqFISH	Sequential barcoding scheme	Dramatically improved throughput	Large number of designed probes needed Optical crowding Long workflow
	MERFISH	Error-robust encoding schemes achieved by introducing a certain Hamming distance	Further improved throughput Error detection and correction	Large number of designed probes needed Optical crowding Long workflow
	corrFISH	Cross-correlating the images from 2 of out of a total of 3 rounds of hybridization	Decode high copy number RNAs	Large number of designed probes needed Long workflow
	seqFISH+	Using secondary probes to dilute target transcripts	Diluting the density of mRNA	Large number of designed probes needed Long workflow
Sequencing-based methods that do not validate cell types	tomo-seq	Cryosectioning of tissue and performing RNA-seq on individual thin sections	Genome-wide RNA tomography of tissues or organs	Often results in 1D data along one axis Often only useful for elongated samples
	Slide-seq Slide-seqV2	DNA-barcoded beads surface	Near-cellular spatial resolution High throughput	No tissue imaging
	sci-Space	Labeling nuclei using unmodified DNA oligos on glass slides	Single-cell resolution Large capture area	Imaging restricted to nuclei
	Stereo-seq	DNA nanoball (DNB) barcoded solid-phase RNA capture	Nano-level resolution (Subcellular) Large capture area	Imaging restricted to nuclei
Sequencing-based methods that validate cell types	LCM-seq	Applying laser energy to remove the cells of interest under microscopy	Speed, precision, and versatility Suitable for a small number of cells	Low throughput Damages adjacent cells RNA contamination
	Geo-seq	Combination of LCM and scRNA-seq	Speed and precision Suitable for a small number of cells	Low throughput Damages adjacent cells RNA contamination
	NICHE-seq	Cells sorted to scRNA-seq after fluorescent markers photoactivated by 2-photon laser scanning microscopy	Single-cell level RNA-seq	Low throughput Reliant on transgenic mice Not suitable for clinical samples
	ST 10X Visium	Barcoded solid-phase RNA capture	Combining histological staining Easy to perform Requires no special instruments Available commercial kit	Low spatial resolution Limited capture area
	HDST	Barcoded solid-phase RNA capture	Improved spatial resolution	Limited capture area
	DBiT-seq	Microfluidic-based method to deliver barcodes to the surface of a tissue slide	Co-mapping of mRNAs and proteins	Limited capture area Spatial resolution needs further improvement
	Seq-Scope	Barcoded solid-phase RNA capture	Nano-level resolution (Subcellular) Large capture area	Needs more research for demonstration
Sequencing-based methods in vivo	TIVA	Photoactivatable mRNA capture in live cells and tissues	Compatible with live and intact tissue, mRNA captured with precise spatial resolution, with little bias from RNA contamination or experimentally-related injury	Low throughput
In situ sequencing methods	FISSEQ	Based on padlock probes and RCA, fixing the cDNA fragments to the cellular protein matrix by amine cross-linker	Preserve the tissue architecture for RNA localization studies, localize subcellular RNA transcriptome-wide	Specific instruments required Random priming is inefficient and may introduce bias Large number of designed probes needed
	STARmap	Based on padlock probes and RCA, integrating hydrogel-tissue chemistry, targeted signal amplification, and In situ SEDAL sequencing	3D volumes of intact tissue, no intrinsic limit to the number of genes	Large number of designed probes needed Workflow is complicated
Methods for FFPE tissue	Smart-3SEQ	Combination of LCM and Smart-3SEQ	Suitable for small samples or those whose RNA is degraded	Needs more research for demonstration
	Visium FFPE	Probes capture RNA after tissue decrosslinked	For clinical samples from biobanks, immunological staining can be performed	Needs more research for demonstration

corrFISH = correlation FISH, FFPE = formalin-fixed paraffin-embedded, FISH = fluorescence in situ hybridization, HDST = high-definition spatial transcriptomics, LCM = laser-capture microdissection, MERFISH = multiplexed error-robust FISH, scRNA-seq = single-cell RNA-sequencing, smFISH = single-molecule FISH, seqFISH = sequential barcoded FISH, SRM = super-resolution microscopy, ST = spatial transcriptomics, TIVA= transcriptome in vivo analysis, DBiT-seq =deterministic barcoding in tissue for spatial omics sequencing, ExFISH=expansion microscopy of fluorescence in situ hybridization, FISSEQ=fluorescent in situ RNA sequencing, Geo-seq=geographical position sequencing, RCA= rolling circle amplification..

## 2. METHODS AND ADVANCES

The core, underlying principle of SRT, involves the acquisition of gene expression profiles while retaining spatial information. Broadly speaking, SRT methods can be classified into 3 groups: imaging-based methods, sequencing-based methods, and in situ sequencing methods. Imaging-based methods are carried out using a microscope, and the transcriptomes are read out through fluorescence in situ hybridization (FISH). Sequencing-based methods are performed using next-generation sequencing technology, and RNA with spatial information is captured and subsequently sequenced. In situ sequencing methods are the combination of imaging- and sequencing-based methods. These methods have their intrinsic strengths and limitations, with differences in target coverage, resolution, and throughput. Like all methods, they must be appropriately matched to the biological question at hand.

### 2.1. Imaging-based methods

Spatial information about protein expression can be obtained from traditional immunohistochemistry (IHC) approaches, which are mediated by the specific binding reaction between antigens and antibodies. In a variation of the IHC method, spatial information about gene expression can be obtained by identifying the mRNA in cells using in situ hybridization (ISH), which involves a hybridization reaction between labeled nucleotide probes and complementary targeting RNA molecules. The single-molecule FISH (smFISH) method is the gold standard for detecting transcripts, and efforts to develop multiplex FISH at the transcriptome level are underway. This would allow FISH to answer more complex biological questions.

#### 2.1.1. smFISH: the gold standard for transcript detection

**Figure 1.**
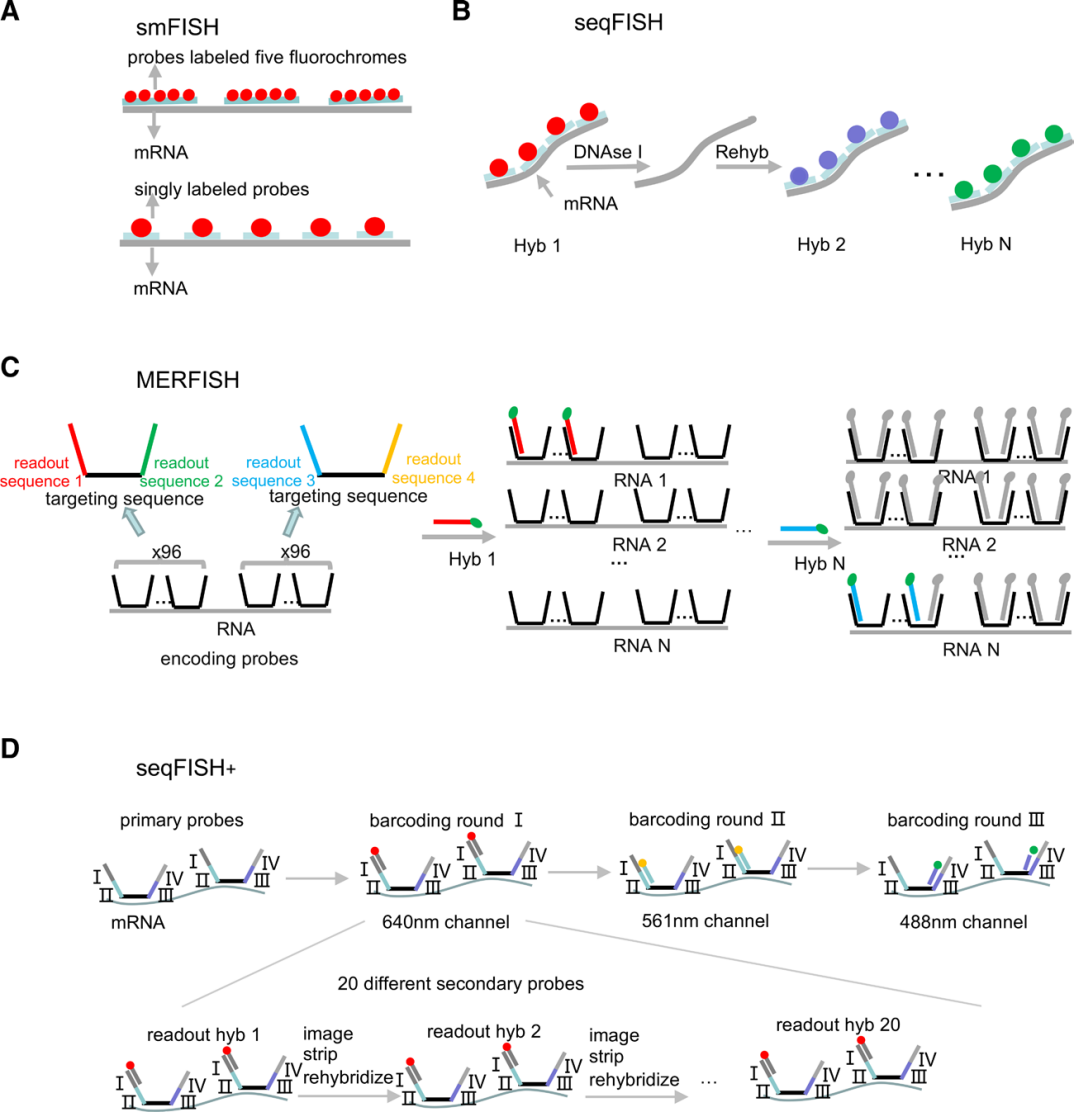
Schematics of the main imaging-based methods. (A) In smFISH, fluorochrome-labeled probes detect individual mRNA molecules through in situ hybridization. (B) In seqFISH, sequential barcoding: in each round of hybridization, a set of probes targeting different genes are hybridized on each transcript, imaged, and then stripped with DNAse I. The same probe sequences are used in different rounds of hybridization, but probes are coupled to different fluorophores. By decoding the temporal color sequences, the original genes can be identified. (C) In MERFISH, HD4 code is introduced by encoding probes containing a central RNA-targeting region flanked by 2 readout sequences. Encoding probes convert the RNA into a unique combination of readout sequences. (D) In seqFISH+, I–IV sequences on the primary probes correspond to 4 rounds of barcoding, and the fourth round is used for error correction. Only one-twentieth of the total genes in each fluorescent channel are labeled by readout probes in each hybridization readout round, lowering the density of transcripts in each image. HD4 = minimum Hamming distance is 4, MERFISH = multiplexed error-robust FISH, seqFISH = seqFISH = sequential barcoded FISH, smFISH = single-molecule FISH.

smFISH combines quantitative FISH with digital imaging microscopy to sensitively detect single RNA molecules using probes. To amplify the signals, probes are often labeled with multi-fluorochromes per molecule.^9^ However, the disadvantages, which include difficulty in acquiring heavily labeled oligonucleotides, the easy loss of coupling fluorophores and self-quenching, highly variable signals associated with multi-fluorophores limit the system to a narrow range of applications. To overcome these drawbacks, singly labeled probes, which are easier to generate and purify, were introduced to accurately detect individual mRNA molecules with uniform signals^10,11^ (Fig. 1A).

smFISH can not only visualize the transcripts in isolated cells or in situ tissues at a high resolution and with high hybridization efficiency, but also provides information about the distribution of subcellular RNA within cells. This feature means that extra analyses concerning subcellular mRNA localization and its functional stages can be achieved, as mRNA localization is currently believed to contribute to the localized regulation of gene expression.^12–15^ In addition, smFISH can precisely measure the copy numbers of specific RNAs without amplification bias and natural fluctuations in gene expression can be quantitatively measured, permitting the elucidation of the mechanisms that regulate gene expression fluctuations and their role in a variety of biological processes. These features make smFISH a robust tool, and it is the standard method used for detecting transcripts.

#### 2.1.2. Multiplexed FISH: improving target coverage

Many biological questions require transcription information about multiple genes, so efforts have been made to address the challenge of the limited number of RNA species that be simultaneously detected in cells using FISH. Super-resolution microscopy (SRM) can resolve a large number of mRNAs in single cells by labeling mRNAs with unique combinations of fluorophores. Although this expanded the scalability of FISH from a few genes to about 30, it was hard to make further progress due to the limited number of fluorophores.^16^ Later, a sequential barcoding scheme was introduced to multiplex different mRNAs, called sequential barcoded FISH (seqFISH).^17^ Here, the mRNA is barcoded by sequential rounds of hybridization, imaging, and probe stripping. The number of RNA species scales as *F^N^*, where *F* is the number of fluorophores and N is the number of rounds of hybridization. Thus, the scalability of seqFISH can be dramatically improved by increasing the number of rounds of hybridization, despite the limited number of fluorophores. In this manner, the entire human transcriptome can be covered, including both mRNAs and non-coding RNAs (Fig. 1B). However, N rounds of hybridizations bring not only exponentially increased throughput, but also exponentially increased detection error rates. When N increases, the error rates increase rapidly, and the majority of RNA molecules are likely to be misidentified after 16 rounds of hybridization.^18^ Therefore, multiplexed error-robust FISH (MERFISH) method was designed. MERFISH is based on combinatorial labeling and sequential imaging, and uses error-robust encoding schemes to correct labeling and detection errors. The method introduces a certain Hamming distance to encode RNAs, and the minimum Hamming distance is 4 (HD4 code), meaning that at least 4 bits must be read incorrectly to change one codeword into another. As a result, every single-bit error produces a word that is uniquely close to a single codeword, allowing such errors to be detected and corrected. In addition, MERFISH improves the multiplexity of transcripts that can be obtained in single cells to 100 to 1000 through the use of binary sequential barcodes^19,20^ (Fig. 1C).

#### 2.1.3. Optical crowding: the major challenge for transcriptome-level profiling

Although seqFISH makes scaling to the level of the genome theoretically possible, transcriptome-level profiling in cells is hindered by optical crowding due to the high density of global transcripts, even when combined with SRM. Some progress has been made to improve the resolution of microscopy, with expansion microscopy (ExM) using a swellable polyelectrolyte gel that can expand physically to magnify the sample. RNAs are covalently attached to the gel, a small molecule linker, and FISH (ExFISH) is performed in cultured cells and intact tissues with high yield and specificity.^21^ Another approach called correlation FISH (corrFISH) was used in seqFISH to read out individual RNA species and barcodes. The principle is simple: RNA species are decoded such that each RNA species appears in only 2 out of a total of 3 rounds of hybridization. By cross-correlating the images from the 2 rounds of hybridization, only the specific RNA species will generate a positive correlation. Although some algorithms have been adapted due to the need for a high degree of temporal resolution, corrFISH still represents a robust method to decode high copy number RNAs in highly multiplexed seqFISH experiments, using conventional fluorescence microscopy.^22^

Another way to address the issue of molecular crowding is to dilute the density of the mRNA. Thus, seqFISH+ labels the secondary probes with fluorophores rather than the primary probes, which provide target sites for the secondary probes. The primary probes divide the transcript set into 20 subsets, and the secondary probes, which are labeled with three colors, ultimately make it possible to visualize one-sixtieth of the transcripts per image in each round of hybridization (Fig. 1D). seqFISH+ achieves transcriptome-level profiling not only for cultured cells but also in tissues, so that unbiased identification of cells alongside their spatial organization can be realized.^23^

### 2.2. Sequencing-based methods

With the development of genetic techniques, next-generation sequencing technology has become a powerful tool for transcriptome analysis. scRNA-seq can precisely characterize cell types and states at the molecular level, without bias, based on global gene expression profiles.^24^ Generally speaking, sequencing-based methods are performed to capture or barcode the RNA before reverse transcription to ensure each transcript can be mapped to its original spatial spot, or are used to gain gene expression information from cells in a certain spatial context. RNA-seq can be conducted either ex situ or in situ.

#### 2.2.1. Sequencing-based methods that do not validate cell types

**Figure 2.**
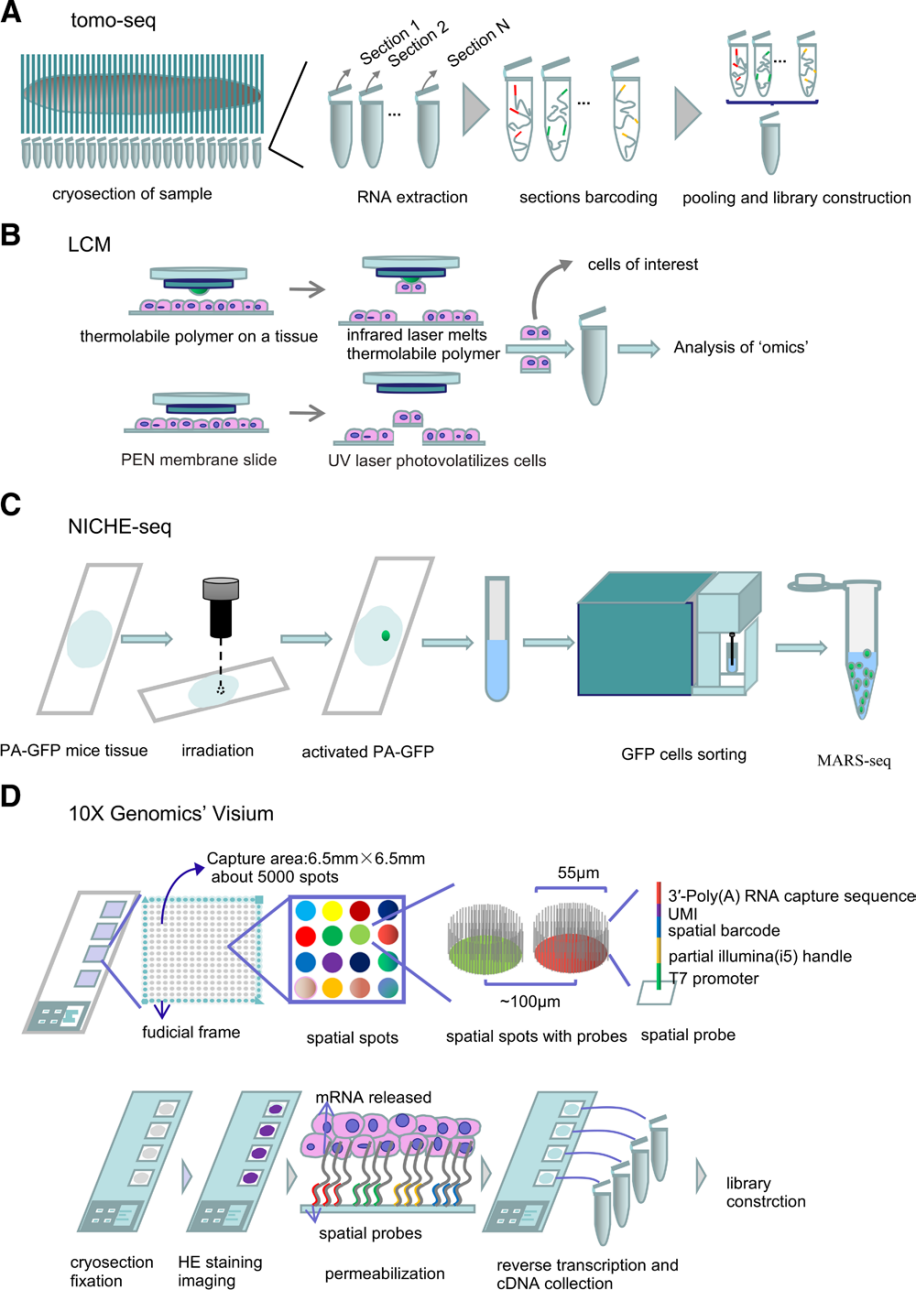
Schematics of the main sequencing-based methods. (A) In tomo-seq, specimens are cryosectioned, and sections are collected in individual microtubes for spatially resolved transcriptomic analysis. (B) In LCM, an infrared laser melts the thermolabile polymer on a tissue section on a glass slide in the vicinity of the laser pulse, resulting in the removal of polymer-cell composite from the tissue. A UV laser can cut away cells of interest or ablate unwanted tissue, leaving cells of interest intact on the slide. (C) In NICHE-seq, tissue expressing a PA-GFP can be activated by 2-photon irradiation, allowing precise in situ labeling. Activated cells are sorted to perform MARS-seq. (D) In 10X Genomics’ Visium, tissue sections are placed on a barcoded glass slide containing 4 capture areas, each with around 5000 spatial spots. After HE staining and imaging, tissue permeabilization releases the mRNA and it is captured by spatial probes. LCM = laser-capture microdissection, PA-GFP = photoactivatable green fluorescent protein, MARS-seq =massively parallel scRNA-seq, UV=ultraviolet, HE staining = hematoxylin-eosin staining.

Some methods do not provide the spatial resolution of microscopy-based techniques as they do not predict or validate cell types using IHC or ISH. Examples of such methods include tomo-seq and Slide-seq.^25,26^ Tomo-seq is a serial microtomy-based sequencing method, which cryosections the tissue of interest and performs RNA-seq on individual thin sections (Fig. 2A). RNA extraction is performed for each individual section and the RNA is barcoded with section-specific primers. This approach can provide genome-wide RNA tomography of tissues or organs, but it is often only possible to cryosection tissue in 1 direction, resulting in 1-dimensional data along 1 axis of the body. For this reason, tomo-seq is best suited for elongated samples. Cells with complex organizations in the same individual sections cannot be analyzed well, and often several identical samples are required to perform cryosectioning in different directions along the main body axes to achieve 2- and 3-dimensional resolution.^27^

Unlike tomo-seq, Slide-seq is a method for transferring RNA from tissue sections onto a surface covered in DNA-barcoded beads with known positions, allowing the locations of the RNA to be inferred by sequencing. It provides a scalable method for obtaining spatially resolved gene expression data at resolutions comparable to the sizes of individual cells.^26^ Slide-seqV2 is an optimized version of Slide-seq with improvements in library generation, bead synthesis, and array indexing to reach an RNA capture efficiency ~10-fold greater than Slide-seq.^28^ A new method, called sci-Space, adapts the low-cost sci-Plex procedure for labeling or “hashing” cell nuclei using unmodified DNA oligos to glass slides. The hashed oligos spotted onto the glass slides are transferred onto permeabilized nuclei (not intact cells) in freshly frozen tissue sections. As the nuclei are stained with 4',6-diamidino-2-phenylindole (DAPI) and the spatial coordinates are consequently imaged during oligo transfer, sci-Space can retain a single-cell resolution.^29^ Obviously, the main limitation of all these methods is that the cell types are purely identified by RNA-seq datasets, which lacks the cell-validation process that microscopy-based techniques provide.

#### 2.2.2. Sequencing-based methods that validate cell types

Although single-cell RNA-seq has the power to define cell types or populations based on gene expression programs, microscopy-based techniques such as IHC or ISH—which validate candidate genes with high cell type specificity—remain the gold standard to define cell type. Thus, a series of sequencing-based methods combined with imaging have been developed to aid the study of solid cell types.

Laser-capture microdissection (LCM) is an optional method to obtain targeted cell subgroups or even single cells quickly and precisely under the microscope. LCM directly harvests cells of interest or isolates specific cells by cutting away unwanted cells via infrared capture systems or ultraviolet cutting systems^30^ (Fig. 2B). Histological staining or rapid antibody staining are performed on the tissues before LCM so that information about the types of cells captured is validated. The key advantages of LCM are that the positional information of cells is maintained and tissues are not dissected; in this way, subsequent analysis by genomics, transcriptomics, or proteomics can be performed on the LCM-captured cells.^31–33^ The extracted nucleic acids can be amplified when the captured regions of interest are small, overcoming the requirement for relatively large numbers of cells. This advantage makes it possible to use LCM at the single-cell level, meaning that it is especially useful for scarce tissues and rare cell types. Geo-seq is the combination of LCM and scRNA-seq technology.^33^ It is also practical to combine LCM with Smart-seq2, a high-throughput scRNA-seq technology with improved sensitivity, accuracy, and full-length coverage across transcripts, making LCM plus Smart-seq2 a promising prospect for SRT.^31,34,35^ However, the main obstacles to the wide application of LCM SRT are its low throughput and potential RNA contamination from other cells, as well as the inevitable damage to adjacent tissue. This means that it is difficult to study the interplay between individual cells and the microenvironment.

One method with similarities to LCM coupled with Smart-seq2 is NICHE-seq, which couples with scRNA-seq. This technique combines photoactivatable fluorescent markers, 2-photon laser scanning microscopy and flow cytometry-based fluorescence-activated cell sorting (FACS), and is coupled to massively parallel scRNA-seq (Fig. 2C). NICHE-seq requires transgenic mice that ubiquitously express a photoactivatable green fluorescent protein (PA-GFP). Two-photon irradiation is used to activate PA-GFP and in situ labeling. After tissue dissociation, GFP-labeled cells can be sorted to perform scRNA-seq; thus, this method combines information about the cell’s transcriptional state and spatial information.^36^ As this method requires transgenic mice expressing GFP, however, it is not suitable for clinical samples.

Another method based on barcoded glass slides, called spatial transcriptomics (ST), also validates cell types by histological staining and imaging.^37^ RNA is spatially captured from tissue sections and barcoded via a microarray on a glass slide. Several primers with unique barcodes are placed on the microscopic glass slide to create a microarray, and a section of tissue is placed on top of the microarray surface. This tissue is then fixed, then stained and imaged, before being permeabilized to release RNA within the cells. In this way, transcripts from the tissue are allowed to meet with the immobilized cDNA synthesis primers in a reverse transcription reaction. Consequently, the cDNA library is sequenced and the data are visualized together with a high-resolution histological image of the tissue section. Each slide consists of about 1000 spots, and ST is both easy to perform and requires no special instruments. The method has been acquired and optimized by 10x Genomics and a commercial kit is available (Fig. 2D).

**Figure 3.**
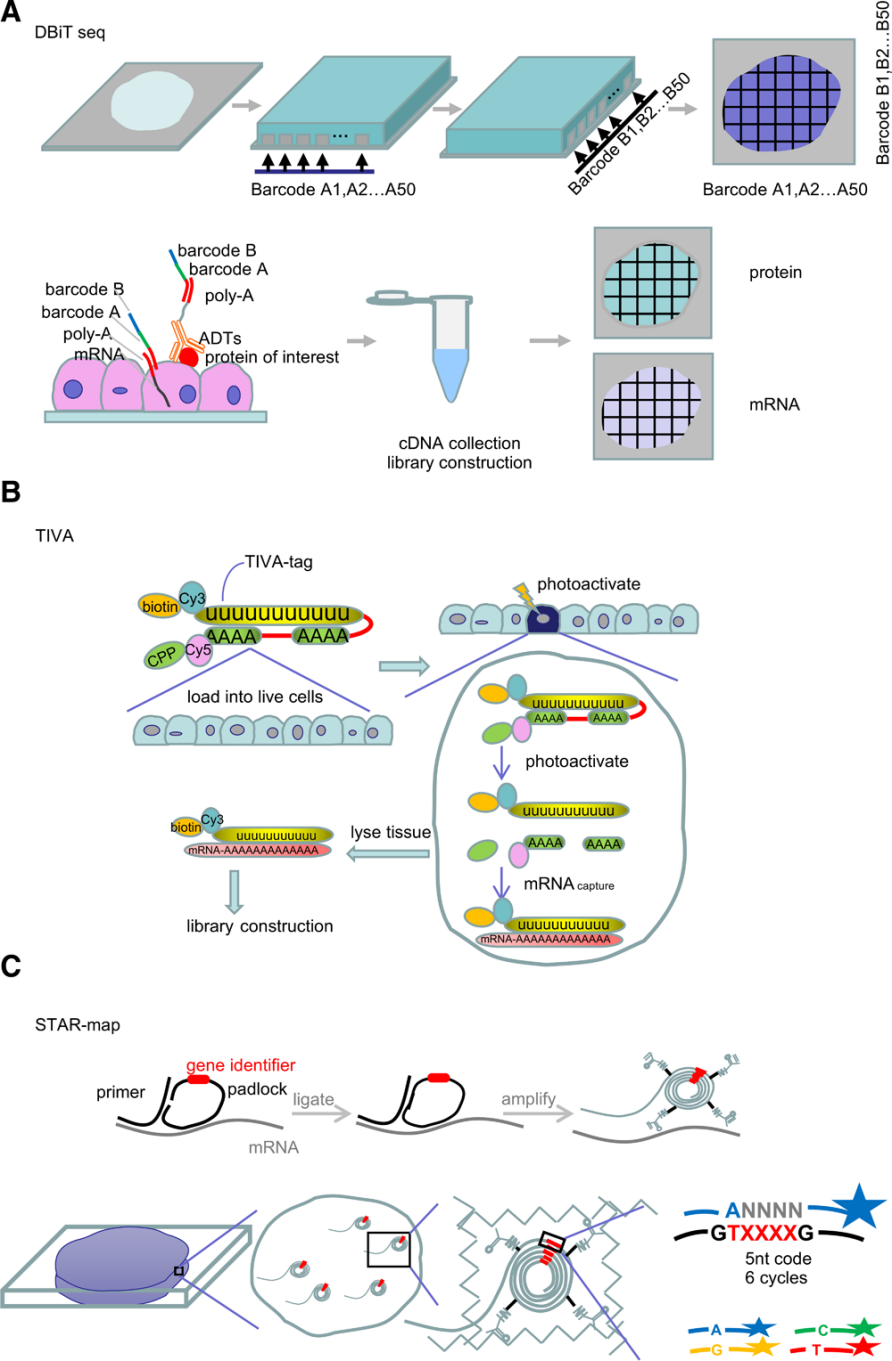
Schematics of DBiT-seq, TIVA, and STAR map. (A) In DBiT-seq, parallel microfluidic channels are used to deliver DNA barcodes to the surface of a tissue slide and yield a 2D mosaic of tissue pixels. ADTs recognize a panel of proteins of interest, co-mapping mRNAs and proteins. (B) In TIVA, caged TIVA-tags are loaded into cells or tissue by virtue of a disulfide-linked CPP. Selective photoactivation of the TIVA-tags in the desired cell or cells using a laser uncages the TIVA-tags, and the exposed poly(U) can anneal to the poly-A tail of cellular mRNA. (C) In STAR map, only when both probes (SNAIL probes) hybridize to the same RNA molecule can the padlock probe be circularized and rolling-circle-amplified to generate a DNA nanoball. The amplicons with an acrylic acid *N*-hydroxysuccinimide moiety modification are copolymerized with acrylamide to embed within a hydrogel network. Gene identifiers are sequenced by in situ SEDAL sequencing. ADTs = antibody-derived DNA tags, CPP = cell-penetrating peptide, STAR = spatially-resolved transcript amplicon readout mapping, TIVA= transcriptome in vivo analysis, DBiT-seq =deterministic barcoding in tissue for spatial omics sequencing, SEDAL=sequencing with error-reduction by dynamic annealing and ligation.

DBiT-seq is a microfluidic-based method that delivers barcodes to the surface of a tissue slide to allow for spatial omic sequencing. This method enables the co-mapping of mRNAs and proteins on a slide containing formaldehyde-fixed tissue^38^ (Fig. 3A). Immunofluorescence can be performed on the same tissue slide, so that proteomics can be integrated with transcriptomics. The capacity for integration is of great significance for multi-omics research.

#### 2.2.3. Sequencing-based methods for use in vivo

Current methods that capture RNA, such as LCM, cannot isolate mRNA from individual in vivo cells without damaging adjacent tissue, making it difficult to assess the influence of the microenvironment on the transcriptome. To overcome this problem, a method was engineered to extract mRNA from live cells or tissues at the spatial resolution of a single cell. This method is called transcriptome in vivo analysis (TIVA); it uses a photoactivatable biotinylated tag, known as a TIVA-tag, that can penetrate the cell membrane by virtue of a cell-penetrating peptide. Once inside the cell, photoactivation is used to cleave the linkers between the poly(U) sequence and two poly(A) stretches to enable the poly(U) sequence to bind the poly-A tail of mRNAs (Fig. 3B). With this method, mRNA can be noninvasively captured within live cells and intact tissues, so it provides a useful tool to explore the transcriptomes of single cells in the context of their natural microenvironment. However, the method is low throughput with regard to the numbers of cells that can be analyzed.

#### 2.2.4. Enhanced resolution and a larger area permit the acquisition of more detail

Solid RNA-barcoding is favorable in terms of its untargeted features and high coverage compared with imaging-based methods. Efforts have been made to improve the spatial resolution and capture area of this method, as a higher resolution means the precise location of the transcripts within the tissues, cells or even subcellular organelles can be determined.

ST can achieve a resolution of only 100 μm (which is not at the single-cell level), which means that the 100 μm spatial spots, with a center-to-center distance of 200 μm, typically cover 5 to 100 cells each, depending on tissue type and region.^37^ Visium technology from 10X Genomics has improved the ST method by reducing the diameter of the capture area from 100 to 55 μm, increasing the capture area to 42.25 mm^2^ (6.5 mm × 6.5 mm), and decreasing workflow duration (Fig. 2D). Nevertheless, the spatial resolution of 10X Genomics’ Visium is still insufficient to resolve single cells in most samples.

Slide-seq and DBiT-seq both have improved spatial resolution to 10 μm,^26,38^ but this is still a near-cellular resolution and cannot be described as single-cell resolution. High-definition spatial transcriptomics (HDST), an optimized version of ST, was designed to improve ST resolution to the single-cell level. In this method, hundreds of thousands of barcodes are randomly deposited onto a slide at 2 μm resolution, and their positions are decoded by sequential hybridization and error-correcting strategy.^39^ One method, called spatiotemporal enhanced resolution omics-sequencing (Stereo-Seq), involves the deposition of a DNA nanoball containing random barcoded sequences to form the chip. Each spot is approximately 220 nm in diameter, with a center-to-center distance of 500 nm.^40^ Another method, known as Seq-Scope, has a center-to-center resolution of 0.5 to 0.8 μm (0.6 μm on average).^41^ The nano-level distances of these methods are far less than the diameter of single cells, giving them the high resolution needed to determine the subcellular location of transcripts.

### 2.3. In situ sequencing methods

The combination of imaging and sequencing processes typically involves in situ sequencing methods. In situ sequencing methods sequence cDNA amplicons based on padlock probes and rolling circle amplification.^42,43^ The 2 ends of the padlock probe can be ligated to form a circle-like structure upon hybridization, which then can be amplified using an isothermal DNA polymerase. Amplification causes copies of the padlock probe sequence to accumulate and form cDNA nanoballs. An amplified signal can be obtained by visualizing the cDNA nanoballs using fluorescently labeled detection oligonucleotides, which are complementary to the padlock probe.

FISSEQ, a highly multiplexed subcellular in situ RNA sequencing method, uses random hexamer-primed reverse transcription in the presence of aminoallyl dUTP. Aminoallyl dUTP then fixes the cDNA fragments to the cellular protein matrix by a non-reversible amine cross-linker.^44^ The templates are circularized after degrading the RNA, and subsequently amplified using rolling circle amplification primers complementary to the adapter sequence introduced by RT. The amplicons in the cells are then ready for sequencing-by-ligation (SOLiD sequencing) at room temperature, and imaging on a confocal microscope. To control the signal density of FISSEQ, a partition sequencing strategy was devised using pre-extended sequencing primers.^44,45^ With 1 base extended, one can randomly sample amplicons at 1/4th of the original density, and with 2 bases extended sampling at 1/16th of the density is possible. One notable highlight of FISSEQ is that it preserves the tissue architecture for RNA localization studies, and localizes subcellular RNA transcriptome-wide. However, the random priming component is inefficient and may introduce bias via uneven binding efficiency, which is due to differences in base composition.

While SRT can be performed on tissues or sections on a slide, applying in situ sequencing methods to 3-dimensional volumes of intact tissue is not easy. To achieve this goal, a method known as spatially resolved transcript amplicon readout mapping (STARmap) integrates hydrogel-tissue chemistry, targeted signal amplification and in situ sequencing.^46^ The method bypasses the reverse transcription step and uses the SNAIL approach instead, which achieves specific amplification of nucleic acids via intramolecular ligation using a pair of custom primer and padlock probes. Only when both probes hybridize to the same RNA molecule can the padlock probe be circularized and rolling-circle-amplified to generate a DNA nanoball. The cDNA amplicons are modified and copolymerized in situ to embed within a hydrogel network. In situ SEDAL sequencing was devised specifically for STARmap, to sequence 5-base barcoded gene-specific identifier segments in SNAIL probes over 6 rounds of hybridization (Fig. 3C). The highlight of STARmap lies in its ability to be used in 3-dimensional in situ transcriptomics, which is undoubtedly a state-of-the-art SRT technique and serves to deepen our understanding of anatomy and transcriptomics. In parallel, there is no intrinsic limit to the number of genes or RNA species that can be simultaneously and quantitatively accessed with STARmap, since it can be adapted to longer sequencing lengths or higher gene numbers.

In conclusion, ISH is highly sensitive, and can detect transcripts at a subcellular resolution. Many powerful strategies have been designed to improve the target coverage of ISH to the transcriptome level and tackle the problem of optical crowding. However, such methods are typically limited by long image acquisition times and a complex probe-designing process. While next-generation sequencing technology enables high-throughput transcriptomic profiling, many sequencing-based methods are much easier to perform once suitable experimental platforms have been established. As spatially barcoded beads or spots can be arrayed more and more densely, some solid-barcoded methods can reach a cellular or subcellular resolution, and spatial information is more precisely retained. To some extent, in situ sequencing methods are a combination of imaging- and sequencing-based methods, sequencing gene identifiers in the designed primers via FISH. Although in situ sequencing methods are high throughput, the complexity of designing targeted probes cannot be ignored (Table 1).

Most of the in situ-based methods described above rely on the use of fresh tissues. However, fresh clinical material is often difficult to obtain for research purposes. To remove the barriers to clinical research, some methods have been designed to use formalin-fixed paraffin-embedded (FFPE) tissue, in which the RNA is degraded. Smart-3SEQ enables large gene expression profiling experiments to be conducted on even small amounts of total RNA, and effectively characterizes small samples extracted by LCM from FFPE tissue.^47^ In addition, 10X Genomics markets a commercially available system, called Visium FFPE, that enables SRT to be performed on samples from a biobank. Some studies have already successfully used 10x Visium FFPE on FFPE tissue.^48^

## 3. DATA ANALYSIS IN SRT

The additional dimension of spatial information brings not only a novel perspective on the transcriptome, but also significant challenges for data analysis due to increased data volume and complexity. Generally speaking, strategies for SRT data analysis include computational approaches specifically designed for SRT and integration methods (with bulk or single-cell RNA-seq data). Different analytical approaches have been developed for localized gene expression pattern identification, spatial decomposition, gene imputation, and cell–cell communication.^49^

Gene expression and spatial location are both of great relevance to biological functions. By analyzing the relationship between gene expression and spatial location, some approaches, including Trendsceek,^50^ SpatialDE,^51^ SPARK,^52^ SPARK-X,^53^ sepal,^54^ SpaGCN,^55^ and GLISS, are designed to identify localized gene expression patterns and spatially variable genes (SVGs) based on different algorithms. Another way to discover SVGs is spatial clustering. Unlike standard clustering methods for scRNA-seq, spatial clustering is for the capture locations. Methods like stLearn, MULTILAYER,^56^ BayesSpace,^57^ SC-MEB,^58^ and STAGATE can be applied for spatial clustering. As most of the sequencing-based methods do not guarantee that a single capture spot contains RNA from only 1 cell, a capture location is often a mixture of multiple cell types. Thus, some spatial decomposition algorithms, such as NNLS, spatialDWLS,^59^ SPOTlight,^60^ RCTD,^61^ DSTG, Tangram, and Cell2location attempt to infer the proportions of cell types in each spot, or score a spot for how strongly it corresponds to a single cellular subtype based on bulk or single-cell RNA-seq data. However, certain SRT methods, particularly those that are imaging-based, cannot provide deep transcriptomic information due to limited gene coverage. Gene imputation provides a chance to impute the missing genes from scRNA-seq data in order to improve the quality of SRT data. Methods that include gene imputation functions include Tangram,^62^ gimVI, Harmony,^63^ LIGER,^64^ Seurat,^65^ SpaGE,^66^ and stPlus.^67^ In addition, the reconstruction of spatial information for scRNA-seq data can be achieved by mapping, which leverages a small set of landmark genes to map the single cell back to its context. Methods that incorporate a mapping function include Harmony, LIGER, Seurat, SpaGE, DEEPsc,^68^ DistMap, SpaOTsc,^69^ novoSpaRc,^70^ and CSOmap.^71^ In addition, cell–cell interactions can be analyzed using some standard algorithms, based on the ligand–receptor interaction pairs from scRNA-seq data and a database of known ligand–receptor interactions. However, the loss of spatial information in scRNA-seq data may lead to inaccurate detection of ligand–receptor interactions, as cellular cross-talk is often spatially restricted. Therefore, SRT data might potentially evaluate the reliability of the ligand–receptor interactions computed from scRNA-seq. In this manner, integrating scRNA-seq with SRT data may accurately reveal specific cell subpopulations and their interactions. Approaches to study cell–cell interactions include SVCA,^72^ GCNG, NCEM, MISTy, stLearn, Squidpy, novoSpaRc, SpaOTsc, and DEEPsc. Among these methods, comprehensive benchmark studies are needed to help users select methods that best fit their data and hypotheses. According to a study benchmarking 16 integration methods, Tangram, gimVI, and SpaGE outperformed other integration methods when predicting the spatial distribution of RNA transcripts, while Cell2location,^73^ SpatialDWLS, and RCTD were the top-performing methods for the cell type deconvolution of spots.^74^

In conclusion, SRT and scRNA-seq data complement each other, since SRT methods cannot always maintain single-cell resolution with the same depth and whole transcriptome coverage of scRNA-seq. The integration of scRNA-seq and SRT data could improve cell type annotation, cell clustering, spatial decomposition, gene imputation, and spatial location reconstruction. A scRNA-seq database could form a solid foundation for SRT data analysis. However, the proper handling of spatial information obtained from SRT data remains a challenge in terms of data processing, and the establishment of proper protocols will be an essential step to reach the full potential of these methods.

## 4. APPLICATIONS FOR SRT

**Figure 4.**
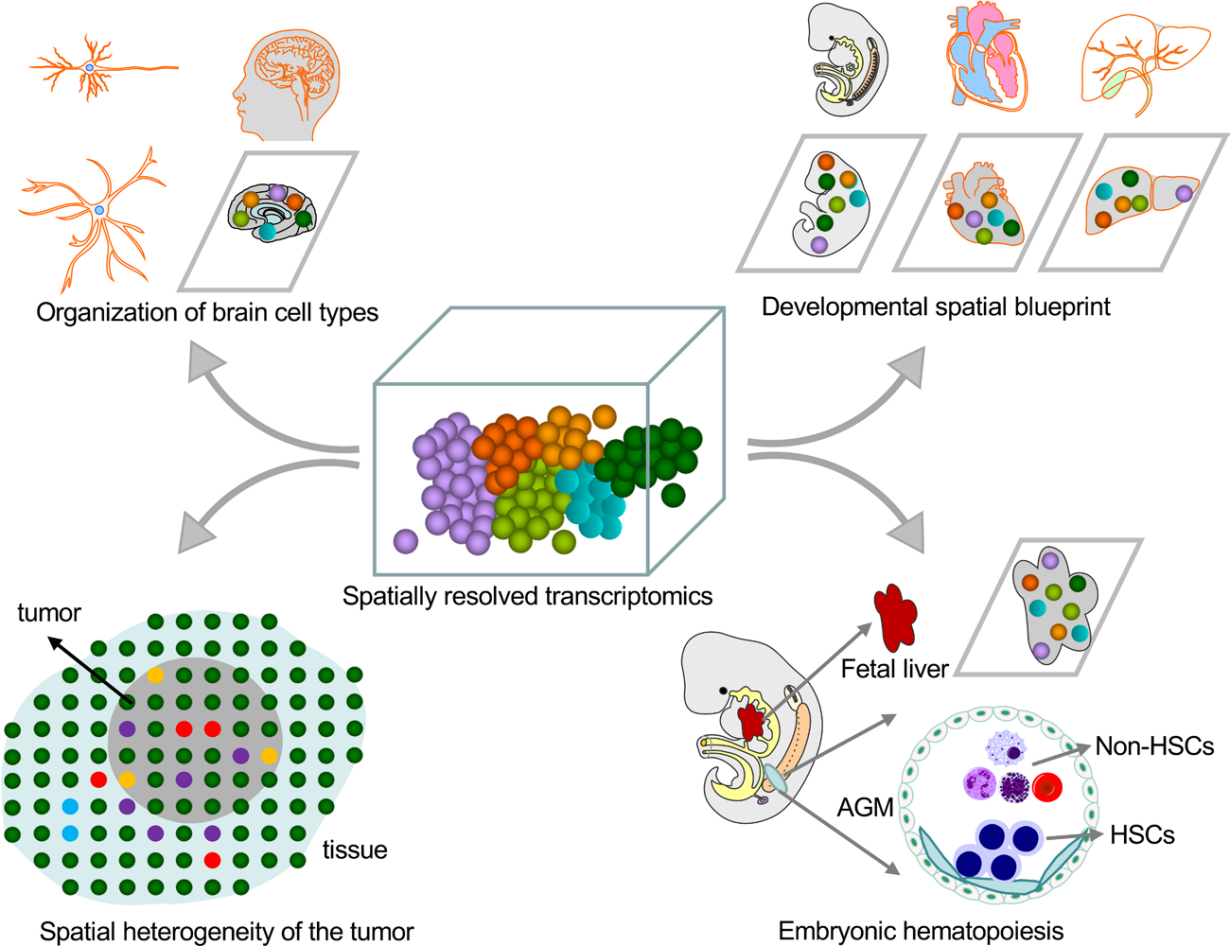
Applications for SRT. In neuroscience, SRT was used to unravel the organization of brain cell types and functions. In cancer biology, the spatial heterogeneity of tumors and the tumor microenvironment can be characterized. In developmental biology, SRT was used to characterize the temporal and spatial expression blueprint of embryonic development. In hematology, most studies utilizing SRT concern embryonic hematopoiesis, and aim to clarify the distinct waves of developmental hematopoiesis. AGM = aorta-gonad-mesonephros, HSCs = hematopoietic stem cells, SRT = spatially resolved transcriptomics.

At present, SRT technologies are widely utilized in basic and clinical research, especially in neuroscience, cancer biology, and developmental biology. However, there are likely further applications for SRT that are yet to be fully utilized, including in the study of hematopoeisis. We reviewed the potential applications in this field in Figure 4.

### 4.1. Neuroscience: unraveling the organization of brain cell types and functions

The diversity of brain cell types and the complex organization of the brain have long been major challenges to our understanding of brain functions during physiological and pathological conditions.^75^ A census of brain cell types and their functions will pave the way for systematic research and, potentially, even novel clinical treatments. SRT has advantages over scRNA-seq due to the retained anatomical and spatial contexts, and various spatially resolved transcriptomic methods have been applied to map cell types in neuroscience. For example, an atlas of the mouse somatosensory cortex was built using osmFISH, a semiautomated version of smFISH. Based on scRNA-seq data, the study identified 31 clusters of cell types and 33 cell type-specific marker genes.^76^ Although multiplexing in osmFISH is relatively low, its sensitivity is higher than scRNA-seq and can provide single-cell-level spatial resolution for gene detection. Multiplexed FISH techniques, such as MERFISH and seqFISH, are applied when more genes need to be analyzed. The multiplexing of 250 genes using seqFISH identified distinct subregions of the hippocampus that contained different combinations of cell types.^18^ In addition, MERFISH combined with measurements of immediate early gene expression identified ~70 neuronal populations in the mouse hypothalamic preoptic area, and defined discrete cell populations activated by specific social behaviors.^77^ STARmap has also been used to reconstruct the 3D locations of cells in the murine primary visual cortex and medial prefrontal cortex; in this particular study, >1000 genes were simultaneously mapped in mouse brain sections at a single-cell resolution.^46^ Sequencing-based methods such as Slide-seq and 10X Genomics’ Visium have also been used to analyze the global transcriptome in both the mouse and human brain.^26,37^

The application of spatially resolved transcriptomic methods is highly likely to improve our understanding of neurological diseases. For example, SRT has already revealed the spatiotemporal dynamics of molecular pathology in amyotrophic lateral sclerosis.^78^ Furthermore, in a murine model of Alzheimer disease, ST and in situ sequencing were used to better characterize the dysregulated cellular network in the early and late phases of disease. The results of this study provided profound insights into the pathogenesis of Alzheimer disease, and also provided clues concerning potential therapeutic targets.^79^

### 4.2. Cancer: profiling the spatial heterogeneity of tumors and the tumor microenvironment

Heterogeneity both within and between tumors is found in most types of cancer.^80,81^ The expansion of tumor subclones with varying genetic alterations and the interactions between tumor cells and the tumor microenvironment presents a complicated situation that influences disease development.^82^ Profiling the spatial heterogeneity of tumors and the tumor microenvironment will provide insights into tumor progression and responses to treatment, and will therefore assist in the diagnosis and treatment of this condition. ST technology has been used to identify intertumoral and intratumoral heterogeneity in multiple types of cancer. Furthermore, changes in gene expression during prostate cancer and cutaneous malignant melanoma progression have revealed a detailed landscape of tumor progression and metastases.^83,84^ Several features of the tumor microenvironment in primary liver cancer, including stromal and immune cell distribution, tumor cluster interaction, cancer stem cell-niche diversity and tertiary lymphoid structure composition have been characterized.^85^ Coupling SRT with other technologies also improves its capacity to characterize tumors: for example, when SRT was combined with scRNA-seq, tissue architecture and interactions between cell subpopulations were revealed in pancreatic ductal adenocarcinomas.^86^ A tumor-specific keratinocyte population, immune infiltrates, and heterogeneity at tumor leading edges have also been identified in human squamous cell carcinoma using multimodal analysis, which included scRNA-seq and ST.^87^ The heterogeneity of breast cancer has also been characterized, and several diagnostic and prognostic markers proposed.^88,89^ Additionally, by integrating spatial gene expression and HE-stained images of breast tumor, a deep learning algorithm was developed to predict the spatially resolved transcriptome of a tissue from the images, enabling image-based screening for molecular biomarkers.^90^ To guide clinical treatment, biomarkers associated with beneficial PD-1 checkpoint blockade in non-small-cell lung cancer were identified using digital spatial profiling.^91^ The ST analysis of human bladder cancer also identified an N-Cadherin 2-expressing epithelial cell subpopulation which could be used to predict therapeutic response, which could guide treatment decisions.^92^ In conclusion, these findings provide novel insights into the complex ecosystem of cancer. and have the potential to improve individualized cancer treatments and drug discovery.

### 4.3. Developmental biology: characterizing the temporal and spatial expression blueprint of embryonic development

Embryonic development is a complex process where dynamic changes rapidly occur at the biomolecular level.^93,94^ Lineage tracing and fate mapping are powerful tools in developmental biology, providing a picture of the lineage hierarchy and linking the initial cell position to future fate.^95^ With the emergence of SRT, analysis of both the temporal and spatial aspects of embryonic development can be performed, and a temporal and spatial expression blueprint is eagerly anticipated. In the early embryo, gastrulation is an essential developmental event, leading to the formation of gastruloids, which are aggregates of embryonic stem cells.^96,97^ Applying Geo-seq to the germ layers from pre- to late-stage gastrulation enabled the molecular genealogy of tissue lineages and the continuum of pluripotency states in time and space to be revealed. Furthermore, the molecular determinants that drive lineage specification and tissue patterning were identified.^98^ Tomo-seq and scRNA-seq revealed genome-wide gene expression patterns in mouse gastruloids and embryos, and identified various embryonic cell types that were not previously known to be present in gastruloids. Based on their results, the study’s authors proposed that somitogenesis occurs in gastruloids.^99^ In addition, ISH has determined the gene expression pattern in gastruloids generated from embryonic stem cells, to validate an in vitro model of early anteroposterior organization during human development.^100^ Stereo-seq has also reconstructed the spatially resolved developmental trajectories of cell-fate transitions and molecular changes during zebrafish embryogenesis.^101^

Some research has also focused on the formation of organs in embryonic development. The large field-of-view and cellular resolution of Stereo-seq has been used to map the spatiotemporal transcriptomic dynamics during mouse organogenesis.^102^ ST was also used to study cardiac morphogenesis, and the spatiotemporal organ-wide gene expression and cell atlas of the developing human heart was established during 3 developmental stages.^103^ Morphogenesis of the human intestine across time, location, and cellular compartments has been charted by both scRNA-seq and 10X Genomics’ Visum.^104^ The gene expression profile of fetal livers from 8 to 17 weeks post-conception in humans has also been illustrated using 10X Genomics’ Visium.^105^

### 4.4. Hematopoiesis: shedding light on the distinct waves of developmental hematopoiesis, and making breakthroughs in adult bone marrow

Hematopoiesis is the process by which blood cells are continually replenished throughout the lifetime of an organism, by virtue of the self-renewal and differentiation of hematopoietic stem cells (HSCs) and the regulation of the local microenvironment, or niche.^106^ Distinct waves of hematopoiesis have been defined during embryogenesis, including both primitive and definitive hematopoiesis.^107,108^ SRT technologies have been applied in developmental hematopoiesis to explore the generation and expansion of HSCs, as well as the regulation of their niche. Geo-seq, combined with bulk and single-cell RNA-seq, was used to examine the caudal hematopoietic tissue of zebrafish (the counterpart of the mammalian fetal liver) and a detailed spatiotemporal transcriptome of hematopoietic stem and progenitor cells (HSPCs) and niche during HSPCs expansion was characterized.^109^ Meanwhile, LCM-seq has been used in the human embryonic aorta-gonad-mesonephros (AGM) region to identify secretory factors that promote human HSC development.^110^ Another study combined with 10X Genomic’s Visium and Stereo-seq identified novel HSC/MPP pocket-like units (HSC PLUS) composed of niche cells and enriched with growth factors; providing an essential resource for understanding HSC/MPP development in the fetal liver.^111^ In addition, 10X Genomic’s Visium was used to visualize HSC emergence in human embryonic tissues, including the liver, AGM, gut, and vitelline and umbilical vessels, validating the generation of AGM-like definitive HSPCs from human pluripotent stem cells.^112^

scRNA-seq technologies have been widely performed in the adult hematopoietic system, and have confirmed the heterogeneity of HSPCs.^113^ However, SRT is yet to be applied in this field. Profiling the SRT of adult bone marrow would be of great significance because this will provide an opportunity to study the interplay between HSPCs and their niche under both normal conditions and stress. Most SRT methods require an intact slice of tissue, which may be challenging for adult bone marrow due to the presence of calcified bone. Therefore, tissue preparation and cryosectioning need to be optimized. Furthermore, bone marrow is composed of various hematopoietic cells and non-hematopoietic cells, including mesenchymal stromal cells, pericytes, fibroblasts, and endothelial cells.^114,115^ Importantly, bone marrow hematopoietic cells are usually smaller than other cell types, and the cells are usually distributed evenly in the bone marrow, lacking any apparent structure in terms of anatomical or functional division.^116,117^ These intrinsic features of bone marrow impede the experimental applications of SRT, as well as associated data processing. LCM-seq has been applied to study adult bone marrow to reveal bone marrow niche organization, setting an excellent example for future research.^118^ RNA-barcoding methods with improved resolution, such as Slide-seq, HDST and stereo-seq, are recommended for bone marrow, since the cellular or subcellular resolution is the key to compensating for smaller cells and diverse cell types. Furthermore, different types of HSPCs share high similarities in transcriptomics. Thus, we propose that SRT data can be properly analyzed only when combined with scRNA-seq data. Of note, in the past decade, single-cell transcriptomic data of bone marrow hematopoietic cells and niche cells has been collected, serving as a supporting resource for SRT.^114,115^

## 5. CONCLUSIONS AND FUTURE PERSPECTIVES

SRT technologies provide an opportunity to uncover the molecular architecture of tissues. Methods that are more scalable, have a higher resolution and easier workflows are expected to be developed, and more results produced by SRT should be published soon. For the remainder of this review, we anticipate future developments that will unlock the full potential of SRT in both biological research and the clinic.

### 5.1. Realizing single-cell SRT

The correct association of detected mRNAs and single cells is a great challenge for both imaging- and sequencing-based methods. For most sequencing-based methods, such as Slide-seq and ST,^26,35,37^ the spatial information is retained in the form of differentially barcoded spots. Although some methods, including HDST, Stereo-seq and Seq-Scope, have achieved cellular or subcellular resolution,^39–41^ the spot boundaries do not correspond to the cell boundaries, meaning that transcripts captured in the same spot do not always come from a single cell. Furthermore, it cannot be guaranteed that the 10 μm-thin slices usually obtained during tissue sampling will contain a single layer of cells. This means that the transcripts of all the cells on the Z-axis or the vertical axis of the slide will be captured on the same spot. In imaging-based methods, the lack of cell boundaries makes it hard to associate individual mRNA molecules with the correct cells. In such methods, DAPI-stained nuclei may be the only method to determine cell outlines, especially when different cell types are very close together. In Sci-Space, which leverages sci-Plex to transfer DNA oligos into permeabilized nuclei, DAPI-stained nuclei and spatial coordinates of oligo transfer are co-registered. The nuclei from the tissue on the slide are also extracted to enable sci-RNA-seq.^29^ Although Sci-Space retains a single-cell resolution, it is hard to avoid contamination from adjacent cells. Moreover, the nuclei-assembling strategy may collapse when applied to tissues that contain irregularly shaped cells, such as neurons and glial cells in the brain. Regardless, we expect that more robust approaches will ultimately permit the characterization of the whole transcriptome of single cells. The elaboration of some cytomembrane-outlining strategies, such as immunofluorescent staining or auto-fluorescent reporter animals to visualize the plasma membrane, may help define cell boundaries and realize the potential of single-cell SRT. Existing rapid membrane imaging methods, such as fluorescent probe-based methods, have already been used to facilitate in situ plasma membrane imaging of neurons and erythrocytes in the complex environment of the brain.^119^ However, optimization of in situ plasma membrane imaging in combination with SRT still requires further work.

### 5.2. Integrating spatially resolved multi-omics to advance our understanding of biological systems

SRT is far more than just transcriptomics; rather, the technique is empowered by the retained positional information provided by in situ methods. The locations of cells and the microenvironment play an important role in uncovering cell-to-cell and cell-to-extracellular matrix interactions, and the integration of functional and spatial information is of great benefit in biological studies. Spatially resolved transcriptomic methods provide several invaluable methods that can also be adapted to suit certain other in situ omics. We expect that SRT will inspire the development of other spatially resolved omics beyond transcriptomics, such as in situ epigenomics, epitranscriptomics, proteomics, and metabolomics, which will help us better understand different aspects of cell function. Epigenomics is a reliable tool to map chromatin accessibility dynamics and higher-order chromatin structure, and adding spatial information to this would surely enable new levels of understanding of cell-fate decisions, identity, and function.^120,121^ Epitranscriptomics concerns a wide range of post-transcriptional RNA modifications, and spatial epitranscriptomics enables studies under specific microenvironments, broadening our understanding of the spatial regulation of gene expression.^122^ In parallel, metabolomics transcends genomics and proteomics, representing the most downstream metabolic stage. The metabolome is widely accepted to constitute a dynamic and sensitive measure of phenotype, placing metabolomics at the forefront of biomarker discovery.^123^ Several spatially resolved metabolomic technologies have already been applied in several fields, such as tumor metabolism, allowing metabolites and metabolic enzymes to be directly discovered and studied in their native state.^124–127^

On the other hand, cell states that are defined based on data from one type of omics may not be accurate and comprehensive, and cell states defined by different omics may not always be in accordance. We therefore expect the emergence of more spatially resolved multimodal technologies, both at the experimental level and in computational analysis, to be able to simultaneously profile multiple data types in the same cell. For example, DBiT-seq can capture both proteins and transcripts on the same sequencing slide, so that proteomics and transcriptomics can be simultaneously performed on the same cells or tissues.^38^ We are also appreciative of and enthusiastic about methods that validate cell types, not just to predict transcriptomic states, but more importantly to provide more possibilities for multi-omic research. For example, some sequencing-based methods with high spatial resolution, such as Stereo-seq, involve the rapid staining and imaging of just the tissue nuclei to avoid mRNA degradation.^40^ Simultaneously retaining the mRNA, protein or metabolites in the same cells may be very challenging. Proper tissue preparation and fixation are essential to preserve the tissue, and so existing workflows containing these steps will need to be optimized to suit multimodal research.

## ACKNOWLEDGMENTS

This work was supported by grants from the Ministry of Science and Technology of China (2021YFA1100900 and 2020YFE0203000), the National Natural Science Foundation of China (81730006, 81922002, 81861148029, and 81870086), CAMS Innovation Fund for Medical Sciences (2021-I2M-1-040 and 2021-I2M-1-019), Haihe Laboratory of Cell Ecosystem Innovation Fund (HH22KYZX0016) and Distinguished Young Scholars of Tianjin (19JCJQJC63400).
